# Fasting Induces Hepatocellular Carcinoma Cell Apoptosis by Inhibiting SET8 Expression

**DOI:** 10.1155/2020/3985089

**Published:** 2020-03-19

**Authors:** Jie Qi, Xiangyuan Chen, Qichao Wu, Jing Wang, Hao Zhang, Anrong Mao, Minmin Zhu, Changhong Miao

**Affiliations:** ^1^Department of Anaesthesiology, Fudan University Shanghai Cancer Center, Department of Oncology, Shanghai Medical College, Fudan University, Shanghai, China; ^2^Department of Hepatic Surgery, Fudan University Shanghai Cancer Center, Department of Oncology, Shanghai Medical College, Fudan University, Shanghai, China

## Abstract

**Background:**

Hepatocellular carcinoma (HCC) is a life-threatening cancer, and the Kelch-like ECH-associated protein 1 (Keap1)/NF-E2-related factor 2 (Nrf2)/antioxidant response element (ARE) signalling pathway plays a crucial role in apoptosis resistance in cancer cells. Fasting is reported to mediate tumour growth reduction and apoptosis. SET8 is involved in cancer proliferation, invasiveness, and migration. However, whether SET8 participates in fasting-mediated apoptosis in HCC remains unclear.

**Methods:**

We used immunohistochemical staining to analyse the expression of SET8, Keap1, and Nrf2 in HCC tissues. Cell viability, apoptosis, and cellular reactive oxygen species (ROS) were assessed, and Western blot and qPCR analyses were used to examine the expression of Keap1/Nrf2 in HCC cells under fasting, SET8 overexpression, and PGC1*α* overexpression conditions. Mass spectrometry, coimmunoprecipitation, and confocal microscopy were used to determine whether PGC1*α* overexpression conditions. Mass spectrometry, coimmunoprecipitation, and confocal microscopy were used to determine whether PGC1*In vivo* experiments were performed to verify the conclusions from the *in vitro* experiments.

**Results:**

Our data indicate that SET8 expression is associated with poor survival in HCC patients. Both *in vitro* experiments. *in vivo* experiments were performed to verify the conclusions from the *α* overexpression conditions. Mass spectrometry, coimmunoprecipitation, and confocal microscopy were used to determine whether PGC1*α* overexpression conditions. Mass spectrometry, coimmunoprecipitation, and confocal microscopy were used to determine whether PGC1

**Conclusions:**

The results of our study demonstrate that fasting induces HCC apoptosis by inhibiting SET8 expression and that SET8 interacts with PGC1*α* to activate the Nrf2/ARE signalling pathway by inhibiting Keap1 expression.*α* overexpression conditions. Mass spectrometry, coimmunoprecipitation, and confocal microscopy were used to determine whether PGC1

## 1. Introduction

The incidence rate of hepatocellular carcinoma ranks sixth among cancers and third for cancer-related mortality worldwide [1]. Resisting apoptosis and sustaining cell growth are recognized as two hallmark features of hepatocellular carcinoma and other cancers [2]. Apoptosis resistance is a major factor responsible for the failure of traditional cancer treatment [3]. Therefore, apoptosis in cancer cells has emerged as a promising target for cancer therapies in hepatocellular carcinoma patients [4].

Fasting, also named dietary restriction or caloric restriction, is a decrease in ad libitum balanced caloric intake by 30% to 60% without causing malnutrition [5]. Cancer is characterized by metabolic dysregulation with increased glucose consumption via upregulation of glycolysis (Warburg effect) and downregulation of oxidative phosphorylation [6]. Fasting is reported to be associated with increased longevity and can provide protection against cancer, cardiovascular disease, diabetes, and cognitive dysfunction [7–10]. Moreover, fasting can reduce tumour growth and induce tumour cell apoptosis [11, 12].

NF-E2-related factor 2 (Nrf2) is a regulator of many genes encoding antioxidant and detoxification enzymes that prevent reactive oxygen species (ROS) accumulation [13]. The stability and accumulation of Nrf2 are modulated by Kelch-like ECH-associated protein 1 (Keap1) [14]. The Keap1/Nrf2/antioxidant response element (ARE) signalling pathway plays a critical role in cellular redox homeostasis. Nrf2 is activated in various types of tumours [15]. Moreover, Nrf2 is abundantly expressed in hepatocellular carcinoma (HCC) cells and is associated with poor HCC prognosis [16].

SET8, also known as SETD8, KMT5A, or PR-Set7, is the only enzyme that generates histone H4 monomethylation on lysine 20 (H4K20me1) in multicellular organisms [17]. SET8 is functional in multiple cellular pathways, such as DNA replication, chromosome compaction, cell cycle progression, transcriptional modulation, genomic instability, and cellular metabolism [17–20]. Moreover, SET8 is involved in cancer proliferation, invasiveness, and migration and is thus associated with a poor survival rate in cancer patients [21, 22]. Some reports show that high methyltransferase activity of SET8 is associated with a high recurrence rate and poor overall survival rate in patients with liver cancer [23]. Consistently, a reduction in SET8 methyltransferase activity increases cellular ROS accumulation [24] and results in massive apoptosis in the epithelium [25]. The role of SET8 in fasting-induced apoptosis in HCC is still not well known. In this study, we investigated the mechanism by which fasting induces HCC cell apoptosis.

## 2. Material and Methods

### 2.1. Clinical Samples

Tumour specimens and paired adjacent liver specimens were randomly collected during surgical resections performed in select patients with HCC at Fudan University Shanghai Cancer Center. The study included a total of 40 participants, each of whom provided informed consent. All the procedures performed in this study were approved by the Ethics Committee of Fudan University.

### 2.2. Analysis of The Cancer Genome Atlas (TCGA) RNASeqV2 Data

TCGA assembly program was used to download hepatocellular carcinoma RNASeqV2 data and clinical data. Cox regression analyses were used to calculate the prognosis in 365 liver cancer patients divided into either the low gene expression group or the high gene expression group of SET8. The overall survival rates of the high and low gene expression groups were compared using Kaplan-Meier analysis and the log-rank test.

### 2.3. Cell Culture and Reagents

MHCC-97H (RRID: CVCL_4972) and HCC-LM3 (RRID: CVCL_6832) cells, which are HCCs, were purchased from the Institute of Biochemistry and Cell Biology, Chinese Academy of Sciences, Shanghai, China. All cell lines were cultured in DMEM containing 1% penicillin-streptomycin and 10% foetal bovine serum at 37°C in a humidified 5% carbon dioxide incubator that was mycoplasma-free.

For starvation experiments, cells were washed with PBS to remove the complete medium and further cultured in DMEM with low glucose (1 mM) and 1% foetal bovine serum, as previously described [26].

### 2.4. Cell Proliferation Assay

The cell counting kit-8 (CCK8) assay (CCK8; Dojindo Molecular Technologies, Inc., Japan) was performed according to the manufacturer's instructions. Cells were plated in 96-well plates at a density of 5 × 10^3^ cells/well. After 24 h, some of the cells were transferred to a fasting concentration (1 mM). Then, 10 *μ*l of CCK8 solution was added to each well, and the cells were incubated with the solution for 2 h. Optical density (OD) values were measured at 450 nm using a microplate reader to indicate the relative cell viability.

### 2.5. Intracellular ROS Detection

Intracellular ROS were measured using a Reactive Oxygen Species Assay Kit (Beyotime Biotechnology) according to the manufacturer's instructions. DCFH-DA (5 *μ*M) was added to the cells, and they were incubated at 37°C for 30 min in the dark. Cells were then washed with serum-free medium three times and analysed for ROS production by flow cytometry.

### 2.6. Apoptosis Assay

Apoptosis was measured by fluorescence-activated cell sorting (FACS) analysis (Cytomics FC 500 MPL; Beckman Coulter, Fullerton, USA) using double staining with Annexin V-FITC and propidium iodide (PI; BD Biosciences, San Jose, USA). Briefly, after different treatments, cells were harvested and incubated with PI and Annexin V-FITC for 30 min at 37°C in the dark and then analysed by flow cytometry.

### 2.7. Immunohistochemistry

Biological specimens were collected as mentioned above. The wax was removed from the tissue slices by washes with PBS, and then, the slices were fixed in 95% alcohol for 30 min and incubated with 3% H_2_O_2_ for 10 min at room temperature to remove endogenous peroxidase activity. Goat serum was added for 10 min at room temperature to block nonspecific staining. Primary SET8 (Abcam, Cambridge, UK), Nrf2 (ProteinTech, 16396-1-AP), and Keap1 (ProteinTech, 10503-2-AP) antibodies were diluted 1 : 200 in the blocking solution and incubated with sections at 37°C for 2 h. Secondary antibody and biotinylated horseradish peroxidase were sequentially added at room temperature for 10 min. Diaminoaniline (DAB) (ZSGB-BIO, China) was added, and samples were counterstained with haematoxylin. Finally, the slides were gradually dehydrated with a graded ethanol series and sealed with neutral glue. Images were acquired with a vertical microscope (Olympus BX53).

### 2.8. Western Blot Analysis

Whole-cell extracts were prepared using cell lysis buffer (Cell Signaling Technology, Danvers, MA). Protein samples were boiled for 5 min in sample loading buffer and were separated by 8-10% SDS-PAGE and transferred to PVDF membranes. Membranes were blocked with 5% skim milk for 1 h and then incubated with primary antibodies overnight at 4°C. The primary antibodies used were as follows: monoclonal antibodies against *β*-actin (ProteinTech, 66009-1-Ig, 1/5000), SET8 (ProteinTech, 14063-1-AP, 1/1000), H4K20me1 (Abcam, Cambridge, UK), Keap1 (ProteinTech, 10503-2-AP, 1/1000), Nrf2 (ProteinTech, 16396-1-AP, 1/1000), Heme oxygenase-1 (HO1) (ProteinTech, 10701-1-AP, 1/1000), Glutamate cysteine ligase subunit catalysis (GCLC) (ProteinTech, 12601-1-AP, 1/1000), Glutamate cysteine ligase modifier subunit (GCLM) (ProteinTech, 14241-1-AP, 1/1000), Malic enzyme 1 (ME1) (ProteinTech, 16619-1-AP, 1/1000), Thioredoxin reductase 1 (TXNRD1) (ProteinTech, 11117-1-AP, 1/1000), and Peroxisome proliferator-activated receptor *γ* coactivator 1*α* (PGC1*α*) (ProteinTech, 66369-1-Ig, 1/1000). After washing the membranes, an HRP-conjugated secondary antibody was then added for 1 h at room temperature, and the membranes were further washed 5 times with TBS-T. Subsequently, the signal was detected by an ECL system. The density of the protein bands was analysed by Scan-gel-it software. Protein expression was normalized to *β*-actin.

### 2.9. Quantitative Polymerase Chain Reaction (qPCR)

Total RNA was isolated from cells by Trizol® reagent (Tiangen Biotech, Beijing, China). cDNA was synthesized using a Hifair® II 1st Strand cDNA Synthesis SuperMix for qPCR (gDNA digester plus) (Yeasen, Shanghai). Quantitative real-time PCR (qPCR) was performed with a Hieff UNICON® qPCR TaqMan Probe Master Mix (Yeasen, Shanghai) to analyse the gene expression of *β*-actin, SET8, Keap1, Nrf2, PGC1*α*, ME1, TXNRD1, GCLC, HO1, and GCLM with a QuantStudio 7 Flex Real-Time PCR System (Applied Biosystems, Life Technologies, Waltham, USA). The qPCR primers used in this study can be found in Table 1.

### 2.10. Immunofluorescence (IF)

Cells were grown on coverslips. After treatment, cells were washed with PBS and fixed in 4% paraformaldehyde for 15 min. After washing 3 times with PBS for 5 min, cells were permeabilized with 0.3% Triton X-100 for 5 min and blocked for 1 h with 1% bovine serum albumin at room temperature. Cells were then incubated with primary anti-SET8 (ProteinTech, 14063-1-AP, 1/200) and anti-PGC1*α* (ProteinTech, 66369-1-Ig, 1/200) antibodies overnight at 4°C. The next day, following washing with PBS, cells were incubated with fluorescent secondary antibodies. After washing 3 times with PBS, cell nuclei were stained with 4,6-diaminophenylindole (DAPI). Images were taken using a confocal fluorescence microscope (Leica).

### 2.11. Coimmunoprecipitation (Co-IP)

Whole cell protein lysates were extracted with a cell lysis buffer with PMSF (Beyotime Biotechnology, Shanghai). For endogenous IP, lysates were incubated with corresponding primary antibodies and 50 *μ*l of protein A/G Dynabeads (Thermo Fisher, USA) at 4°C overnight. Then, 10 *μ*l of input, IgG negative control, and the IP were subjected to Western blotting.

### 2.12. Mass Spectrometry

Cells were transfected with a SET8 plasmid, and protein lysate was extracted 48 h after transfection. The endogenous IP was performed as described above. Silver staining was performed using a fast silver staining kit (Beyotime Biotechnology, Shanghai) according to the manufacturer's instructions. Mass spectrometric analysis of stained gel strips was performed using high-performance liquid chromatography (1260 Series, Agilent Technologies) and mass spectrometry (Agilent 6460, Agilent Technologies).

### 2.13. Chromatin Immunoprecipitation (ChIP)

ChIP assays were carried out with a Simple ChIP Plus Sonication Chromatin IP Kit (Cell Signaling Technology, MA) according to the manufacturer's instructions. Briefly, cells (1 × 10^7^) were fixed with 1% formaldehyde for 10 min at room temperature to cross-link DNA and proteins. Glycine was then added to stop the cross-linking reaction. Chromatin was sheared using a Microson Ultrasonic Cell Disruptor XL (Misonix) with 16 cycles of sonication (15 s each, 2 min rest, amplitude = 10, power = 15 W). Ten microliters of sonicate was collected from each sample as input, and the remaining sample was incubated with anti-PGC1*α* (Abcam, USA) or anti-H4K20me1 (Abcam, USA) antibodies or an IgG negative control at 4°C overnight. Immunoprecipitants were bound to protein G magnetic beads, and the DNA-protein cross-linking was reversed by incubating at 65°C for 2 h. Then, the DNA was purified, and enriched DNA sequences were analysed by qPCR. Keap1 oligonucleotide sequences for PCR primers were as follows: forward 5′-TGACAAAACTGAGCCTCCTAGC-3′ and reverse 5′-GCATCAAAGAGTGATGCTGAATG-3′.

### 2.14. Dual-Luciferase Assay

A Promega Dual-Luciferase Assay Kit (Madison, WI, United States) was used to assess the impact of SET8 and PGC1*α* on Keap1 promoter activity. The Keap1 promoter was amplified from genomic DNA of HCC-LM3 cells and ligated into a pGL3-Basic vector to generate a pGL3-Keap1 construct. pGL3-Keap1 was transfected with a Renilla luciferase vector into HCC-LM3 cells, and the impact of SET8 and PGC1*α* on Keap1 promoter activity was assessed using a dual-luciferase assay kit.

### 2.15. siRNA Treatments

MHCC-97H and HCC-LM3 cells were transfected with siRNA against PGC1*α* using Lipofectamine 3000 (Invitrogen, USA) according to the manufacturer's instructions. The PGC1*α* siRNA sequences (Biotend, Shanghai) were sense, 5′-GCUCCAAGACUCUAGAAdTdT-3′, and anti-sense, 5′-UUGUCUAGAGUCUUGGAGCdTdT-3′; for siRNA #2, the sequences were sense, 5′-GGCAGUAGAUCCUCUUCAAdTdT-3′, and anti-sense, 5′-UUGAAGAGGAUCUACUGCCdTdT-3′.

### 2.16. SET8 Lentivirus Containing Short Hairpin RNAs (shRNAs) and Mutant Treatments

SET8 shRNAs (Genechem, Shanghai) and mutant SET8^R295G^ plasmid were transfected into HCC cells. The shRNA sequences were as follows: shRNA-1, 5′-CAACAGAATCGCAAACTTA-3′; shRNA-2, 5′-CAACAGAATCGCAAACTTA-3′.

### 2.17. Fasting in Mice

All animal studies and procedures adhered to the recommendations of the Medicine and Public Health Animal Care and Use Committee of Shanghai Medical College at Fudan University. Wild-type BALB/c mice (female, 6 weeks, 20–25 g) were purchased from Shanghai Sippr-BK Laboratory Animal Co. Ltd. Mice were divided into four groups based on the interventions (*N* = 6 per group): group 1: mice injected with MHCC-97H control cells; group 2: mice injected with shSET8 knockdown MHCC-97H cells; group 3: mice injected with MHCC-97H cells and fasted; group 4: mice injected with SET8-overexpressing MHCC-97H cells and fasted. Mice in all groups were injected subcutaneously in the right flank with 100 *μ*l of cells in PBS at a density of 2 × 10^6^ cells/ml. Animals were divided into 4 groups at 5-7 days after inoculation of tumour cells. Group 1 and group 2 mice were maintained under standard conditions throughout the study. Group 3 mice, which were injected with MHCC-97H cells, and group 4 mice, which were injected with SET8-overexpressing MHCC-97H cells, were subjected to alternating days of fasting and days of ad libitum diet (on nonfasting days). Animals were given free access to water every day [26]. Mice were individually housed in clean new cages to avoid cannibalism or cofeeding. Tumour size, body weight, and general behaviour were monitored every 4 days. The tumour size was calculated using callipers, and the tumour volume was calculated as follows: tumour volume (mm^3^) = (length × width × width) × *π*/6, where expression length and width are in millimetres. Tumours were harvested and weighed for WB and qPCR analysis.

### 2.18. Statistical Analysis

The results are presented as the mean ± SD (standard deviation). Two-tailed unpaired *t*-tests or one-way ANOVA with GraphPad Prism Version 6 (GraphPad Software, San Diego, CA) was performed to compare the groups. *P* < 0.05 was considered significant.

## 3. Results

### 3.1. SET8 Is Upregulated and Associated with a Poor Prognosis in Hepatocellular Carcinoma

**Figure 1:**
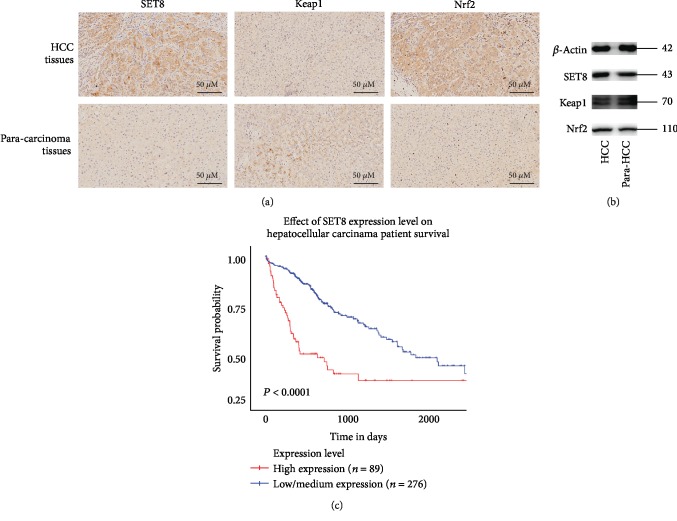
SET8 is upregulated in HCC and is positively associated with a poor prognosis. (a) Immunohistochemical analysis of SET8, Keap1, and Nrf2 expression in HCC tissues and paracarcinoma normal liver tissue specimens from patients with HCC. Nuclei (blue) were stained by haematoxylin. (b) Western blot analysis of SET8, Keap1, and Nrf2 expression in HCC specimens and adjacent paracarcinoma normal liver tissue specimens from patients with HCC. (c) Kaplan-Meier OS survival curve for patients with high or low SET8 expression in TCGA dataset of HCC; ^∗^*P* < 0.05.

First, we analysed the expression of SET8, Keap1, and Nrf2 by immunohistochemical staining in tumour tissues and adjacent nontumour tissues from hepatocellular carcinoma patients who underwent a surgical resection. We found that both Nrf2 and SET8 were highly expressed in HCC tissues in comparison to paracarcinoma tissues, and the staining was mainly restricted to the nucleus. In contrast, Keap1 was more highly expressed in paracarcinoma tissues than in HCC tissues and was mainly present in the cytoplasm (Figure 1(a)). Similar results were found by analysing protein expression by Western blot, which showed that SET8 and Nrf2 expression was higher in HCC tissues than in adjacent nontumour tissues, while Keap1 expression was higher in adjacent tissues (Figure 1(b)). Next, we assessed the overall survival rates of HCC patients using TCGA dataset of HCC. Patients were divided into two groups on the basis of SET8 expression. We found that higher SET8 expression was positively associated with a poorer overall survival rate in patients with HCC (Figure 1(c)).

### 3.2. Effects of Fasting on Cell Viability, Apoptosis, and Expression of Components of the SET8 and Keap1/Nrf2/ARE Signalling Pathways

**Figure 2:**
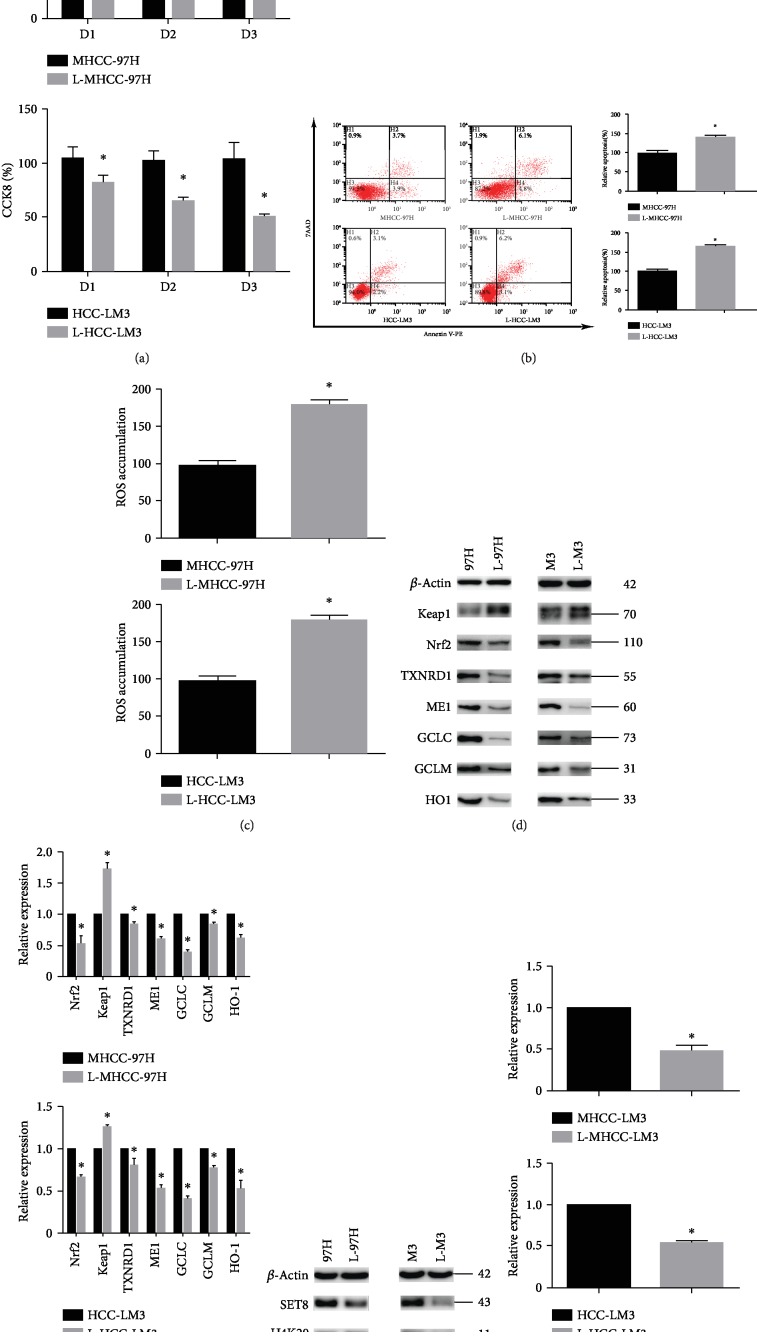
Effects of fasting on cell viability, apoptosis, ROS level, and expression of Keap1/Nrf2/ARE signalling pathway components and SET8. (a) Proliferation of MHCC-97H and HCC-LM3 cells in normal or fasting conditions for 24 h, as measured by CCK8. (b) Flow cytometry was used to detect apoptotic MHCC-97H and HCC-LM3 cells cultured in normal or fasting medium for 24 h. (c) The level of ROS was detected by flow cytometry in MHCC-97H and HCC-LM3 cells cultured in normal or fasting medium for 24 h. (d) Western blot analysis of the Keap1/Nrf2/ARE signalling pathway in MHCC-97H and HCC-LM3 cells cultured in normal or fasting conditions for 24 h. (e) The mRNA expression of the Keap1/Nrf2/ARE signalling pathway components in HCC cells grown in normal or fasting medium for 24 h was examined by qPCR. (f) Western blot analysis of SET8 and H4K20me1 in HCC cells cultured in normal or fasting medium for 24 h. (g) The mRNA expression of SET8 was examined by qPCR in cells grown in normal or fasting medium for 24 h. Data are shown as the mean ± SD of five independent experiments. ^∗^*P* < 0.05 vs. the control group.

MHCC-97H and HCC-LM3 cells were cultured in fasting medium or complete DMEM (control). Compared to the control group, fasting reduced cell viability (Figure 2(a)) and induced apoptosis (Figure 2(b)) in MHCC-97H and HCC-LM3 cells. Moreover, compared with the control group, fasting increased ROS accumulation in HCC (Figure 2(c)). Previous studies have reported that the Keap1/Nrf2/ARE signalling pathway plays a critical role in cellular redox homeostasis. We analysed the expression of the Keap1/Nrf2/ARE signalling pathway components in MHCC-97H and HCC-LM3 cells by qPCR or Western blotting. The results showed that fasting increased the expression of Keap1 but decreased the expression of Nrf2, ME1, TXNRD1, HO1, GCLM, and GCLC at the protein (Figure 2(d)) and mRNA (Figure 2(e)) levels. Furthermore, the mRNA and protein expression of SET8 and its substrate H4K20me1 was decreased under fasting conditions (Figures 2(f) and 2(g)).

To determine the role of Keap1 in response to fasting, we constructed a Keap1 knockdown model by treating HCC cells with Keap1 siRNA under fasting treatments. Keap1 knockdown improved fasting-mediated loss of cell viability and increased apoptosis in HCC cells (Supplementary Figures [Supplementary-material supplementary-material-1]). Furthermore, knockdown of Keap1 counteracted fasting-mediated ROS accumulation in HCC cells (Supplementary [Supplementary-material supplementary-material-1]). Moreover, the additional knockdown of Keap1 led to an increase in the expression of Nrf2/ARE signalling pathway components in fasting-treated HCC cells (Supplementary Figures [Supplementary-material supplementary-material-1]). These data indicated that fasting mediated HCC apoptosis, ROS accumulation, and inhibition of the Nrf2/ARE signalling pathway via upregulation of Keap1 expression.

### 3.3. Role of SET8 in HCC Cell Viability, Apoptosis, ROS Accumulation, and the Expression of Keap1/Nrf2/ARE Signalling Pathway Components in response to Fasting

**Figure 3:**
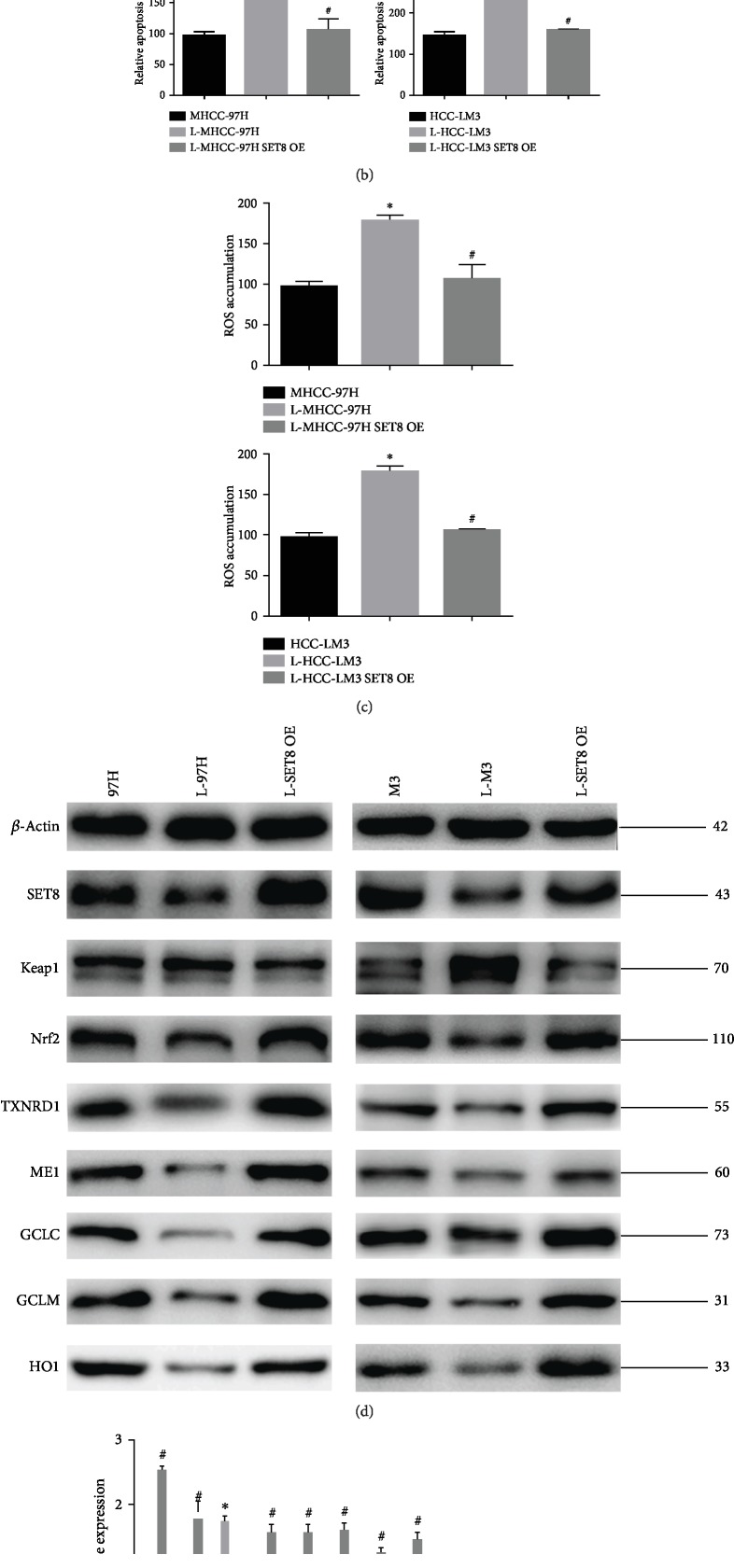
Role of SET8 in HCC cell viability and apoptosis in response to fasting. (a) Proliferation of MHCC-97H and HCC-LM3 cells under fasting conditions for 24 h or fasting in combination with overexpressed SET8. (b) Flow cytometry was used to detect the number of apoptotic MHCC-97H and HCC-LM3 cells under fasting conditions for 24 h or fasting in combination with overexpressed SET8. (c) The level of reactive oxygen species in MHCC-97H and HCC-LM3 cells under fasting conditions for 24 h or fasting in combination with overexpressed SET8. (d) Western blot analysis of SET8 and Keap1/Nrf2/ARE signalling pathway components in MHCC-97H and HCC-LM3 cells under fasting conditions for 24 h or fasting in combination with overexpressed SET8. (e) qPCR analysis of SET8 and Keap1/Nrf2/ARE signalling pathway components in MHCC-97H and HCC-LM3 cells under fasting conditions for 24 h or fasting in combination with overexpressed SET8. (f) ChIP assay of H4K20me1 presence at the promoter region of Keap1. Normal IgG was used as a control. (g) SET8 knockdown increased Keap1 luciferase activity in HCC-LM3 cells. Data are shown as the mean ± SD of five independent experiments. ^∗^*P* < 0.05 vs. the control group. ^#^*P* < 0.05 vs. the fasting group.

To understand the role of SET8 in fasting-mediated decreases in cell viability and increases in apoptosis, SET8 was overexpressed or silenced in MHCC-97H and HCC-LM3 cells. We found that overexpression of SET8 reversed fasting-mediated loss of cell viability (Figure 3(a)) and increased apoptosis (Figure 3(b)). Moreover, knockdown of SET8 resulted in decreased cell viability (Supplementary [Supplementary-material supplementary-material-1]) and increased apoptosis (Supplementary [Supplementary-material supplementary-material-1]) in HCC cells, which was similar to the effects observed with fasting. Furthermore, overexpression of SET8 counteracted fasting-mediated ROS accumulation (Figure 3(c)). Similarly, SET8 knockdown augmented ROS accumulation in HCC cells, which was similar to the effect observed with fasting (Supplementary [Supplementary-material supplementary-material-1]). We then analysed the effect of SET8 on the expression of the Keap1/Nrf2/ARE signalling pathway by qPCR or Western blotting. SET8 overexpression under fasting conditions in HCC cells decreased Keap1 expression, while it increased the expression of Nrf2/ARE signalling pathway components at the protein (Figure 3(d)) and mRNA (Figure 3(e)) levels. Moreover, SET8 knockdown increased the expression of Keap1 and decreased the expression of the Nrf2/ARE signalling pathway in HCC cells, which was similar to the effect observed with fasting (Supplementary Figures [Supplementary-material supplementary-material-1]). These data indicated that fasting induced HCC apoptosis and inhibition of the Keap1/Nrf2/ARE signalling pathway via a decrease in SET8 expression.

Next, to identify whether Keap1 is targeted by SET8, we examined the genome-wide distribution of H4K20me1, a downstream target of SET8, in HCC-LM3 cells by ChIP assay. H4K20me1 was found to enrich at the Keap1 promoter region (Figure 3(f)). Luciferase reporter assays indicated that SET8 knockdown enhanced Keap1 promoter activity (Figure 3(g)).

Then, we demonstrated the role of Keap1 in SET8 knockdown HCC cells. Keap1 was silenced in MHCC-97H and HCC-LM3 cells by Keap1 siRNA. Knockdown of Keap1 by siRNA reversed SET8 knockdown-mediated loss of cell viability (Supplementary [Supplementary-material supplementary-material-1]) and increased apoptosis (Supplementary [Supplementary-material supplementary-material-1]) in HCC cells. Moreover, Keap1 knockdown weakened ROS accumulation in SET8-silenced HCC cells (Supplementary [Supplementary-material supplementary-material-1]). Furthermore, the additional knockdown of Keap1 led to an increase in the expression of Nrf2/ARE signalling pathway components in SET8-silenced MHCC-97H and HCC-LM3 cells (Supplementary Figures [Supplementary-material supplementary-material-1]). These data indicated that SET8 positively regulated Nrf2/ARE signalling pathway expression and HCC malignant potential by inhibiting Keap1 expression.

### 3.4. SET8 Interacts with PGC1

**Figure 4:**
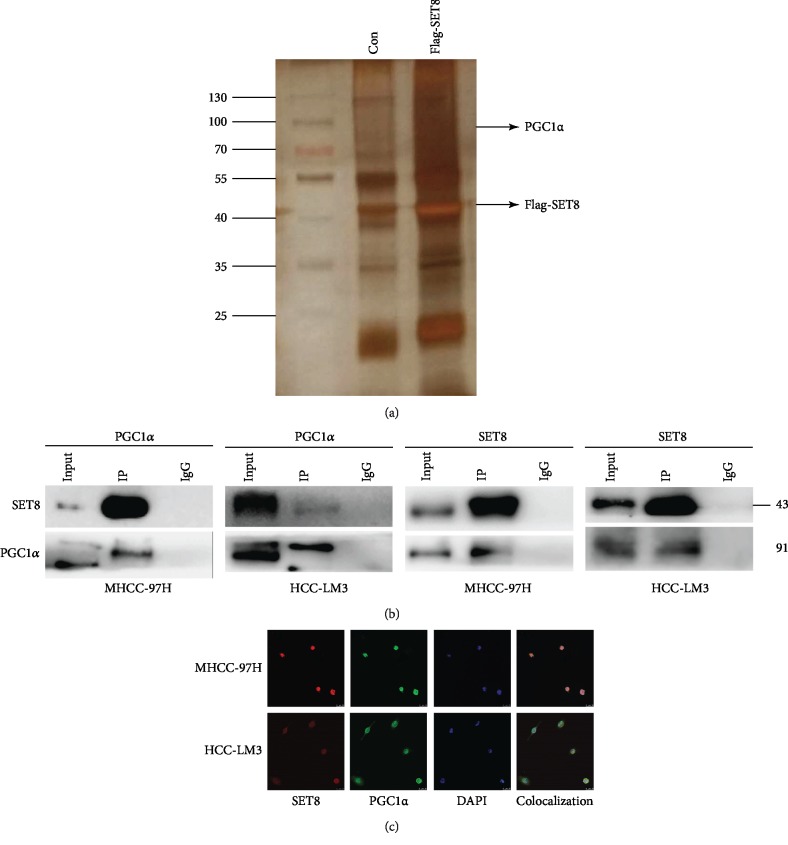
SET8 interacts with PGC1. (a) Mass spectrometry was used to analyse protein bands in the purified SET8 complex. (b) Interaction between SET8 and PGC1*α* in HCC cells was measured by immunoprecipitation. (c) Colocalization of SET8 and PGC1*α* in HCC cells as detected by confocal microscopy.

Purified SET8 complexes were resolved by SDS-PAGE followed by silver staining. The differential protein bands were isolated and analysed by mass spectrometry. Among several other proteins, we found that PGC1*α* probably interacts with SET8 (Figure 4(a)). Furthermore, we verified that SET8 coprecipitated with PGC1*α* in MHCC-97H and HCC-LM3 cells (Figure 4(b)). Finally, we demonstrated by confocal microscopy that SET8 and PGC1*α* colocalized with each other (Figure 4(c)).

### 3.5. Role of PGC1*α* in HCC Cell Viability, Apoptosis, ROS Accumulation, and Expression of Keap1/Nrf2/ARE Signalling Pathway Components in response to Fasting

**Figure 5:**
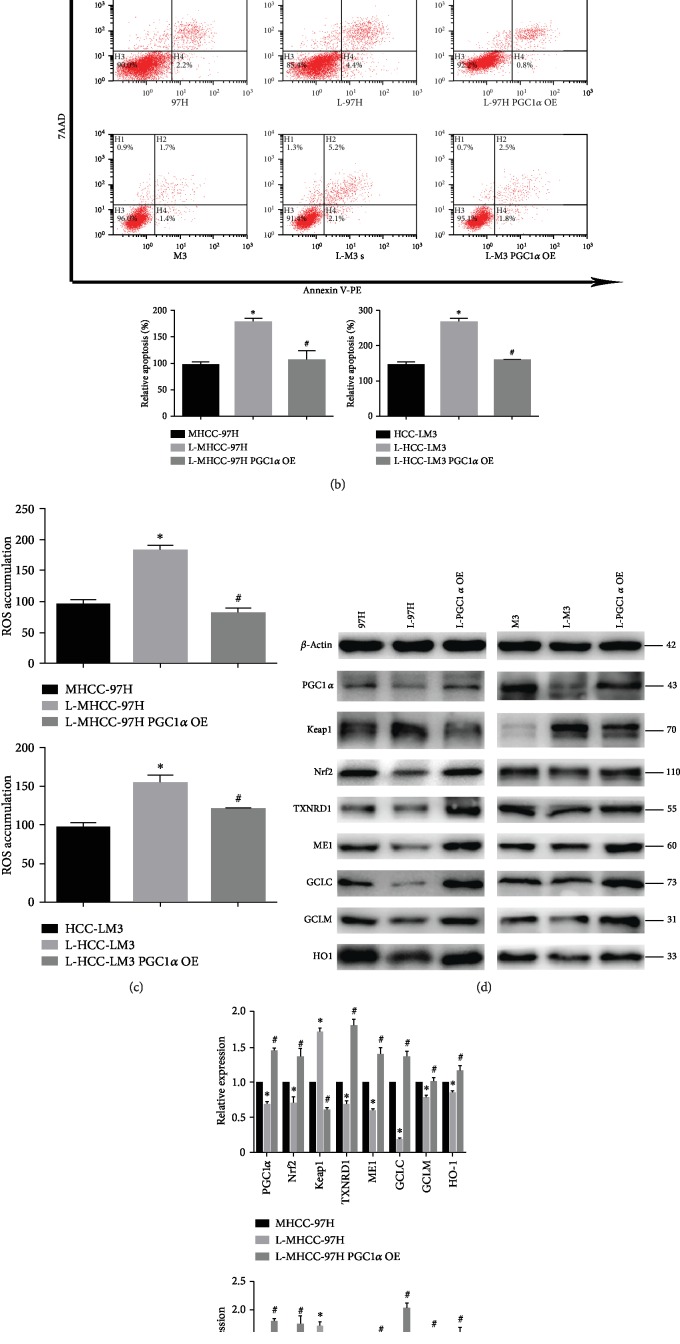
Role of PGC1*α* in HCC cell viability and apoptosis in response to fasting. (a) Proliferation of MHCC-97H and HCC-LM3 cells under fasting conditions for 24 h or fasting in combination with overexpressed PGC1*α*. (b) Flow cytometry was used to detect the number of apoptotic MHCC-97H and HCC-LM3 cells under fasting conditions for 24 h or fasting in combination with overexpressed PGC1*α*. (c) The level of reactive oxygen species in MHCC-97H and HCC-LM3 cells under fasting conditions for 24 h or fasting in combination with overexpressed PGC1*α*. (d) Western blot analysis of PGC1*α* and Keap1/Nrf2/ARE signalling pathway components in MHCC-97H and HCC-LM3 cells under fasting conditions for 24 h or fasting in combination with overexpressed PGC1*α*. (e) qPCR analysis of PGC1*α* and Keap1/Nrf2/ARE signalling pathway components in MHCC-97H and HCC-LM3 cells under fasting conditions for 24 h or fasting in combination with overexpressed PGC1*α*. (f) ChIP assay of PGC1*α* presence at the promoter region of Keap1. Normal IgG was used as a control. (g) PGC1*α* knockdown increased Keap1 luciferase activity in HCC-LM3 cells. Data are shown as the mean ± SD of five independent experiments. ^∗^*P* < 0.05 vs. the control group. ^#^*P* < 0.05 vs. the fasting group.

PGC1*α* was overexpressed or silenced in MHCC-97H and HCC-LM3 cells. We found that overexpression of PGC1*α* reversed fasting-mediated decreases in cell viability (Figure 5(a)) and increases in apoptosis (Figure 5(b)). Moreover, knockdown of PGC1*α* resulted in decreased cell viability (Supplementary [Supplementary-material supplementary-material-1]) and increased apoptosis (Supplementary [Supplementary-material supplementary-material-1]) in MHCC-97H and HCC-LM3 cells, which was similar to the effect observed in fasting cells. Furthermore, overexpression of PGC1*α* counteracted fasting-mediated ROS accumulation in HCC (Figure 5(c)). Similarly, PGC1*α* knockdown augmented ROS accumulation in HCC cells (Supplementary [Supplementary-material supplementary-material-1]), which was similar to the effect observed in fasting cells. We then analysed the effect of PGC1*α* on Keap1/Nrf2/ARE signalling pathway expression by qPCR or Western blotting. PGC1*α* overexpression led to a decrease in Keap1 expression and an increase in Nrf2/ARE signalling pathway expression under fasting conditions (Figures 5(d) and 5(e)). Moreover, PGC1*α* knockdown increased the expression of Keap1 while decreasing the expression of Nrf2/ARE signalling pathway components in HCC cells (Supplementary Figures [Supplementary-material supplementary-material-1]), which was similar to the effects observed in fasting cells.

### 3.6. SET8 Interacts with PGC1*α* to Positively Regulate Keap1 in HCC *In Vitro*, and Fasting Inhibits HCC Growth via SET8 Inhibition *In Vivo*

**Figure 6:**
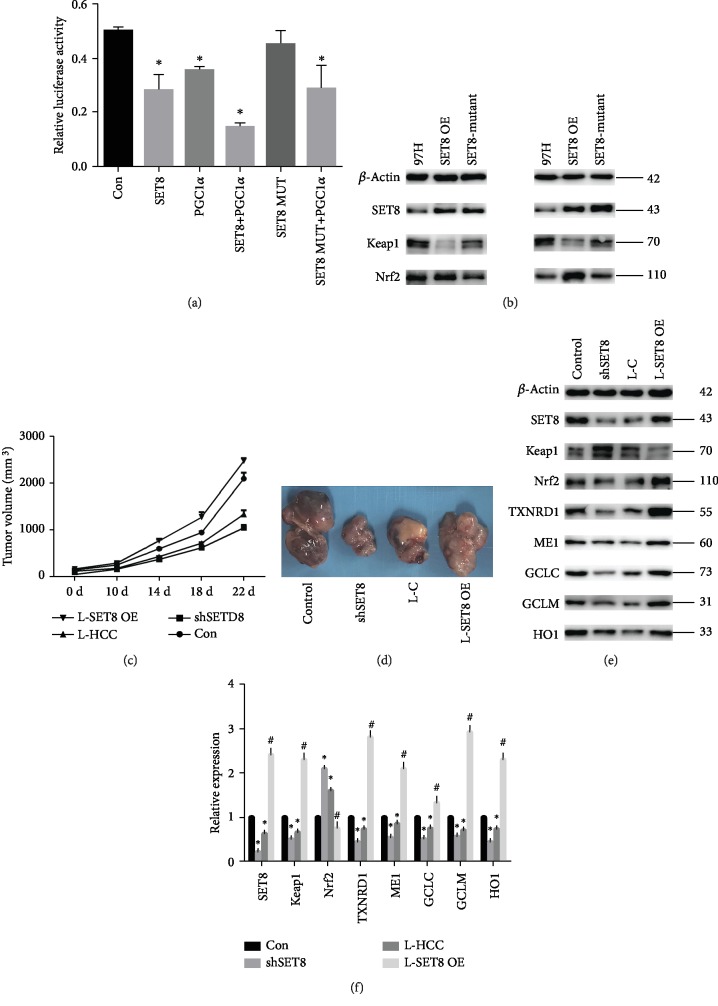
SET8 interacts with PGC1*α* to regulate Keap1 in HCC cells *in vitro*, and fasting inhibits HCC growth via SET8 inhibition *in vivo*. (a) The effects of SET8, PGC1*α*, and mutant SET8 on Keap1 luciferase activity in HCC-LM3 cells. (b) Western blotting was used to detect the SET8, Keap1, and Nrf2 proteins in MHCC-97 and HCC-LM3 cells overexpressing wild-type or mutant SET8. (c, d) Tumour growth following subcutaneous injection in the right flanks of Balb/c nude mice with 97H control cells, 97H-shSET8 cells, or 97H-SET8 overexpression cells (*n* = 6). (e) Western blotting was used to detect proteins SET8 and Keap1/Nrf2/ARE signalling pathway components in mice. (f) The mRNA expression of SET8 and Keap1/Nrf2/ARE signalling pathway components was examined by qPCR *in vivo*. Data are shown as the mean ± SD of five independent experiments. ^∗^*P* < 0.05 vs. the control group. ^#^*P* < 0.05 vs. the fasting group.

Luciferase reporter assays indicated that SET8 not only attenuated Keap1 promoter activity but also strengthened the negative effect of PGC1*α* on Keap1 promoter activity (Figure 6(a)). Moreover, mutant SET8^R295G^ had no effect on Keap1 promoter activity in HCC cells (Figure 6(a)). Furthermore, Nrf2 expression was upregulated, while Keap1 expression was decreased in HCC cells overexpressing SET8 but not in those expressing mutant SET8^R295G^ (Figure 6(b)).

Then, *in vivo* experiments were performed to verify the above conclusion. We found that fasting mice and mice injected with SET8 knockdown HCC cells had repressed tumour growth compared to the control group (Figures 6(c) and 6(d)). Additionally, in these mice, the expression of Nrf2 and its downstream effectors was decreased, while the expression of Keap1 was elevated (Figures 6(e) and 6(f)). We also found that the antitumour effects of fasting could be counteracted by overexpressing SET8 (Figures 6(c)–6(f)).

## 4. Discussion

Mitochondria are the major cellular organelles that generate intracellular ROS and play a key role in apoptosis [27]. Excessive ROS production results in biomolecule damage and cancer cell apoptosis, and ROS have been widely found to play a crucial role in apoptosis upon cancer treatment [28]. Nrf2 is a member of the basic leucine zipper transcription factor NF-E2 family, which coordinates the induction of antioxidant and phase II detoxifying enzymes [29]. Keap1 is a component of the Cullin 3-based E3 ubiquitin ligase complex that controls the stability and accumulation of Nrf2 [30]. Normally, Nrf2 binds to Keap1 in the cytoplasm and is then degraded by the proteasome pathway. Once activated, Nrf2 is translocated to the nucleus and binds to ARE to activate downstream phase II cell protective enzymes, including TXNRD1, ME1, GCLC, GCLM, and HO-1. After translation, the Nrf2 protein is rapidly degraded in the cytoplasm by the ubiquitin-proteasome system [30]. The Keap1/Nrf2/ARE signalling pathway is one of the most crucial antioxidant stress pathways in cells, and it plays an important role in redox regulation and oncogenic pathways [31]. Many studies have established that cancer cells survive under stress conditions via Nrf2-mediated oxidation resistance [32]. The mechanism by which Nrf2 promotes cancer cell formation and progression includes inhibition of apoptosis, induction of detoxification enzymes, and expression of antioxidative stress genes [33]. The activation of cell protective factors downstream of Nrf2 is conducive to the survival of cancer cells and resistance of cancer cells to chemotherapy [34]. It has been reported that Nrf2 is involved in the expression of antiapoptotic proteins, promotes chemotherapy tolerance of liver cancer, and is associated with the expression of Bcl-xl, an antiapoptotic gene [35]. In addition, it has been observed that ME1 expression is positively correlated with larger tumour size, higher grade, poorer survival, and chemotherapy resistance in breast cancer patients [36]. In the present study, knockdown of Keap1 under fasting conditions resulted in enhanced cell viability, the inhibition of apoptosis and ROS accumulation, and increased expression of Nrf2/ARE signalling pathway components (Supplementary Figures [Supplementary-material supplementary-material-1]). These data indicate that fasting mediated HCC apoptosis and Nrf2/ARE signalling pathway inhibition by upregulating Keap1 expression (Figures 2(a)–2(c)).

SET8 is the only enzyme that generates histone H4 monomethylation on lysine 20 (H4K20me1) in multicellular organisms [17]. SET8 plays a key role in the epigenetic regulation of genes in many cellular processes [37]. Higher expression of SET8 in tumours is associated with high recurrence and low overall survival [23]. These observations indicate that SET8 may be a potential therapeutic target for tumour therapy. There is also evidence that SET8 may function as a barrier to mitochondrial oxidative phosphorylation activity [38]. SET8 was found to induce NQO1, a reductase that inhibits inflammation and apoptosis in cells [39]. Our previous study indicated that SET8 aggravated glycolytic metabolism and thus induced HCC progression [40]. Therefore, downregulation of SET8 expression could attenuate the malignant potential of cancer cells. In the present study, we found that SET8 was expressed at higher levels in HCC tissues than it was in paracarcinoma tissues (Figures 1(a) and 1(b)). To investigate the possible role of SET8 in the Keap1/Nrf2/ARE system in HCC, we transfected HCC cells to cause either knockdown or overexpression of SET8. We found that knockdown of SET8 downregulated the expression of Nrf2 and its downstream effectors and upregulated Keap1 expression (Supplementary Figures [Supplementary-material supplementary-material-1]). Moreover, the additional knockdown of Keap1 led to an increase in the expression of Nrf2/ARE signalling pathway components in SET8-silenced HCC cells (Supplementary Figures [Supplementary-material supplementary-material-1]). Furthermore, the ChIP assay in this study revealed that H4K20me1 is enriched in the Keap1 promoter region (Figure 3(f)). Luciferase reporter assays indicated that SET8 knockdown enhanced Keap1 promoter activity (Figure 3(g)), while SET8 overexpression attenuated Keap1 promoter activity (Figure 6(a)). These findings suggest that SET8 negatively modulates Keap1 expression and thus participates in fasting-mediated HCC cell apoptosis.

PGC1*α* is a transcriptional coactivator of several transcription factors and nuclear receptors that regulate mitochondrial biogenesis and mitochondrial function [41]. PGC1*α* is important for rapid cell adaptation to energy-demanding environments because it regulates oxidative phosphorylation, Krebs cycle enzymes, fatty acid oxidation, antioxidant components, and ROS levels [42]. Thus, PGC1*α* is expressed especially in metabolically active tissues such as the liver, kidneys, and brain [43]. The role of PGC1*α* in carcinogenesis has not been clear. Most experts believe early in carcinogenesis, PGC1*α* may be downregulated, while in the later stages of tumour progression, PGC1*α* is often upregulated [44]. A study in mice showed that PGC1*α* regulates mitochondrial function and promotes tumour growth [45]. PGC1*α* is highly upregulated and facilitates cancer metastasis in lung cancer [46]. To investigate whether PGC1*α* affects the Keap1/Nrf2/ARE system in HCC, we transfected HCC cells to either knock down or overexpress PGC1*α*. Our data demonstrated that knockdown of PGC1*α* enhanced apoptosis, mediated ROS accumulation, and inhibited the Nrf2/ARE signalling pathway via upregulation of Keap1 expression. Moreover, PGC1*α* overexpression attenuated fasting-mediated apoptosis and increased the tumour antioxidant capacity (Figures 5(b) and 5(c), [Supplementary-material supplementary-material-1]). Furthermore, ChIP assay results indicated that PGC1*α* was enriched at the Keap1 promoter region (Figure 5(f)). Luciferase reporter assays further indicated that PGC1*α* knockdown enhanced Keap1 promoter activity (Figure 5(g)). These data indicate that PGC1*α* negatively modulates Keap1 expression, thus participating in fasting-mediated Nrf2/ARE signalling pathway inhibition and HCC cell apoptosis. Furthermore, we found that PGC1*α* interacted with SET8 by mass spectrometry analysis (Figure 4(a)). Co-IP and immunofluorescence analysis validated the interaction between and colocalization of PGC1*α* and SET8 (Figures 4(b) and 4(c)). In addition, luciferase reporter assays indicated that SET8 not only attenuated Keap1 promoter activity but also strengthened the negative effect of PGC1*α* on Keap1 promoter activity (Figure 6(a)). These results suggest that SET8 binds to PGC1*α* to attenuate the activity of the promoter of Keap1, which leads to high expression of Nrf2 and a high level of oxidation resistance in HCC cells.

In previous studies, fasting was shown to reduce mammary carcinogenesis and decrease cancer cell proliferation [47, 48]. Another report in 4T1 breast cancer cells showed that the antitumour effect of fasting was mediated by increased oxidative stress and apoptosis [49]. Additionally, caloric restriction is reported to decrease proliferation, increase apoptosis, and decrease the metastatic burden in triple-negative breast cancers [50]. Other studies have observed that a fasting-mimicking diet downregulated the expression of HO-1 [48, 51]. The ketogenic diet is a type of fasting diet, and it can exacerbate metabolic oxidative stress in tumour cells [52]. In this study, we found that fasting increased apoptosis and ROS accumulation and inhibited the Nrf2/ARE signalling system via upregulation of Keap1 expression in HCC (Figures 2(a)–2(e)).

It is well known that fasting is the most physiological means of inducing autophagy [53]. Moreover, a previous study indicated that the inhibitory effect of fasting on tumour growth depends on autophagy [48]. Whether fasting-mediated autophagy was also regulated by SET8 deserves further research.

## 5. Conclusions

In summary, the present study demonstrated that fasting increased apoptosis and ROS accumulation and downregulated Nrf2/ARE signalling pathway expression via upregulation of Keap1 expression in HCC cells. Moreover, fasting inhibited SET8 and PGC1*α* expression in HCC. Furthermore, SET8 interacted with PGC1*α* to negatively regulate Keap1 expression, which participated in fasting-induced HCC apoptosis.

## Figures and Tables

**Table 1:** Primer sequences.

Genes	Sequences	
	Forward (5′-3′)	Reverse (5′-3′)
*β-Actin*	ATGCCCTGAGGCTCTTTTCCAGCC	CCAGGATGGAGCCACCGATCCACA
*SET8*	AGCTCCAGGAAGAGCAAAGCCGAG	GGCGTCGGTGATCTCGATGAGGT
*Keap1*	CACCACAACAGTGTGGAGAGGTA	TACAGTTGTGCAGGACGCAGACG
*Nrf2*	CCAATTCAGCCAGCCCAGCACAT	GGTGACTGAGCCTGATTAGTAGC
*PGC1α*	CGGAAATCATATCCAACCAG	TGAGGACCGCTAGCAAGTTTG
*ME1*	CCTCACTACTGCTGAGGTTATAGC	CGGTTCAGGATAAACTGTGGCTG
*TXNRD1*	GCAATCCAGGCAGGAAGATTGCT	CTCTTGACGGAATCGTCCATTCC
*GCLC*	GTGGTACTGCTCACCAGAGTG	AGCTCCGTGCTGTTCTGGGCCTT
*HO1*	AGCGGGCCAGCAACAAAGTGCAA	CAGCATGCCTGCATTCACATGGC
*GCLM*	ATCTTGCCTCCTGCTGTGTGATGC	CAATGACCGAATACCGCAGTAGCC

## Data Availability

The data that support the findings of this study are available from the corresponding authors upon reasonable request.

## Supplementary Materials

Supplementary Materials Supplementary Figure S1: (A) proliferation of MHCC-97H and HCC-LM3 cells under fasting conditions for 24 h or fasting in combination with Keap1 knockdown, (B) flow cytometry was used to detect the number of apoptotic MHCC-97H and HCC-LM3 cells under fasting conditions for 24 h or fasting in combination with Keap1 knockdown, (C) the level of reactive oxygen species in MHCC-97H and HCC-LM3 cells under fasting conditions for 24 h or fasting in combination with Keap1 knockdown, (D) Western blot analysis of SET8 and Keap1/Nrf2/ARE signalling pathway components in MHCC-97H and HCC-LM3 cells under fasting conditions for 24 h or fasting in combination with Keap1 knockdown, (E) qPCR analysis of SET8 and Keap1/Nrf2/ARE signalling pathway components in MHCC97H and HCC-LM3 cells under fasting conditions for 24 h or fasting in combination with Keap1 knockdown. Data are shown as the mean ± SD of five independent experiments. ^∗^*P* < 0.05 vs. the control group. ^#^*P* < 0.05 vs. the fasting group. Supplementary Figure S2: (A) proliferation of MHCC-97H and HCC-LM3 cells following SET8 knockdown, (B) flow cytometry was used to detect the number of apoptotic MHCC-97H and HCC-LM3 cells following SET8 knockdown, (C) the level of reactive oxygen species in MHCC-97H and HCC-LM3 cells following SET8 knockdown, (D) Western blot analysis of SET8 and Keap1/Nrf2/ARE signalling pathway components in MHCC-97H and HCC-LM3 cells following SET8 knockdown, (E) qPCR analysis of SET8 and Keap1/Nrf2/ARE signalling pathway components in MHCC-97H and HCC-LM3 cells following SET8 knockdown. Data are shown as the mean ± SD of five independent experiments. ^∗^*P* < 0.05 vs. the control group. Supplementary Figure S3: (A) proliferation of MHCC-97H and HCC-LM3 cells following SET8 knockdown alone or in combination with Keap1 knockdown, (B) flow cytometry was used to detect the number of apoptotic MHCC-97H and HCC-LM3 cells following SET8 knockdown alone or in combination with Keap1 knockdown, (C) the level of reactive oxygen species in MHCC97H and HCC-LM3 cells following SET8 knockdown alone or in combination with Keap1 knockdown, (D) Western blot analysis of SET8 and Keap1/Nrf2/ARE signalling pathway components in MHCC-97H and HCC-LM3 cells following SET8 knockdown alone or in combination with Keap1 knockdown, (E) qPCR analysis of SET8 and Keap1/Nrf2/ARE signalling pathway components in MHCC-97H and HCC-LM3 cells following SET8 knockdown alone or in combination with Keap1 knockdown. Data are shown as the mean ± SD of five independent experiments. ^∗^*P* < 0.05 vs. the control group. ^#^*P* < 0.05 vs. the SET8 knockdown group. Supplementary Figure S4: (A) proliferation of MHCC-97H and HCC-LM3 cells following PGC1*α* knockdown, (B) flow cytometry was used to detect the number of apoptotic MHCC-97H and HCC-LM3 cells following PGC1*α* knockdown, (C) the level of reactive oxygen species in MHCC-97H and HCC-LM3 cells following PGC1*α* knockdown, (D) Western blot analysis of PGC1*α* and Keap1/Nrf2/ARE signalling pathway components in MHCC-97H and HCC-LM3 cells following PGC1*α* knockdown, (E) qPCR analysis of PGC1*α* and Keap1/Nrf2/ARE signalling pathway components in MHCC97H and HCC-LM3 cells following PGC1*α* knockdown. Data are shown as the mean ± SD of three independent experiments. ^∗^*P* < 0.05 vs. the control group.
