# Supplementary material for: Fasting Induces Hepatocellular Carcinoma Cell Apoptosis by Inhibiting SET8 Expression
Source: Oxid Med Cell Longev. 2020 Mar 19;2020:3985089. doi: 10.1155/2020/3985089 (PMC7115168; doi:10.1155/2020/3985089)
Supplement: Supplementary Materials — Supplementary Figure S1: (A) proliferation of MHCC-97H and HCC-LM3 cells under fasting conditions for 24 h or fasting in combination with Keap1 knockdown, (B) flow cytometry was used to detect the number of apoptotic MHCC-97H and HCC-LM3 cells under fasting conditions for 24 h or fasting in combination with Keap1 knockdown, (C) the level of reactive oxygen species in MHCC-97H and HCC-LM3 cells under fasting conditions for 24 h or fasting in combination with Keap1 knockdown, (D) Western blot analysis of SET8 and Keap1/Nrf2/ARE signalling pathway components in MHCC-97H and HCC-LM3 cells under fasting conditions for 24 h or fasting in combination with Keap1 knockdown, (E) qPCR analysis of SET8 and Keap1/Nrf2/ARE signalling pathway components in MHCC97H and HCC-LM3 cells under fasting conditions for 24 h or fasting in combination with Keap1 knockdown. Data are shown as the mean ± SD of five independent experiments. ∗P < 0.05 vs. the control group. #P < 0.05 vs. the fasting group. Supplementary Figure S2: (A) proliferation of MHCC-97H and HCC-LM3 cells following SET8 knockdown, (B) flow cytometry was used to detect the number of apoptotic MHCC-97H and HCC-LM3 cells following SET8 knockdown, (C) the level of reactive oxygen species in MHCC-97H and HCC-LM3 cells following SET8 knockdown, (D) Western blot analysis of SET8 and Keap1/Nrf2/ARE signalling pathway components in MHCC-97H and HCC-LM3 cells following SET8 knockdown, (E) qPCR analysis of SET8 and Keap1/Nrf2/ARE signalling pathway components in MHCC-97H and HCC-LM3 cells following SET8 knockdown. Data are shown as the mean ± SD of five independent experiments. ∗P < 0.05 vs. the control group. Supplementary Figure S3: (A) proliferation of MHCC-97H and HCC-LM3 cells following SET8 knockdown alone or in combination with Keap1 knockdown, (B) flow cytometry was used to detect the number of apoptotic MHCC-97H and HCC-LM3 cells following SET8 knockdown alone or in combination with Keap1 knockdown, (C) the level of reactive [file 3985089.f1.pdf]

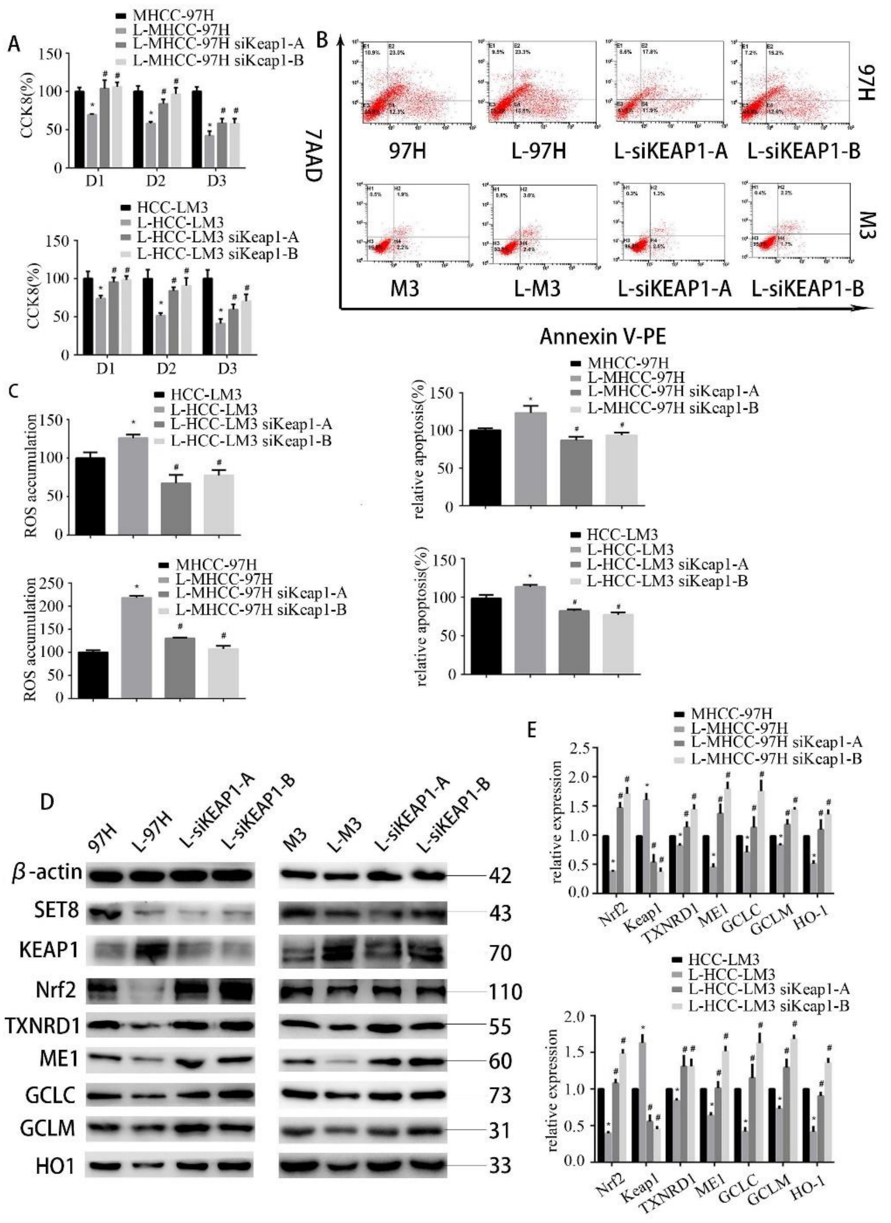

Supplementary Figure S1

(A) Proliferation of MHCC-97H and HCC-LM3 cells under fasting conditions for 24 h or fasting in combination with Keap1 knockdown. (B) Flow cytometry was used to detect the number of apoptotic MHCC-97H and HCC-LM3 cells under fasting conditions for 24 h or fasting in combination with Keap1 knockdown. (C) The level of reactive oxygen species in MHCC-97H and HCC-LM3 cells under fasting conditions for

24 h or fasting in combination with Keap1 knockdown. (D) Western blot analysis of SET8 and Keap1/Nrf2/ARE signalling pathway components in MHCC-97H and HCC-LM3 cells under fasting conditions for 24 h or fasting in combination with Keap1 knockdown. (E) qPCR analysis of SET8 and Keap1/Nrf2/ARE signalling pathway components in MHCC-97H and HCC-LM3 cells under fasting conditions for 24 h or fasting in combination with Keap1 knockdown. Data are shown as the mean ± SD of five independent experiments. *$P < 0.05$ vs the control group. # $P<0.05$ vs the fasting group.

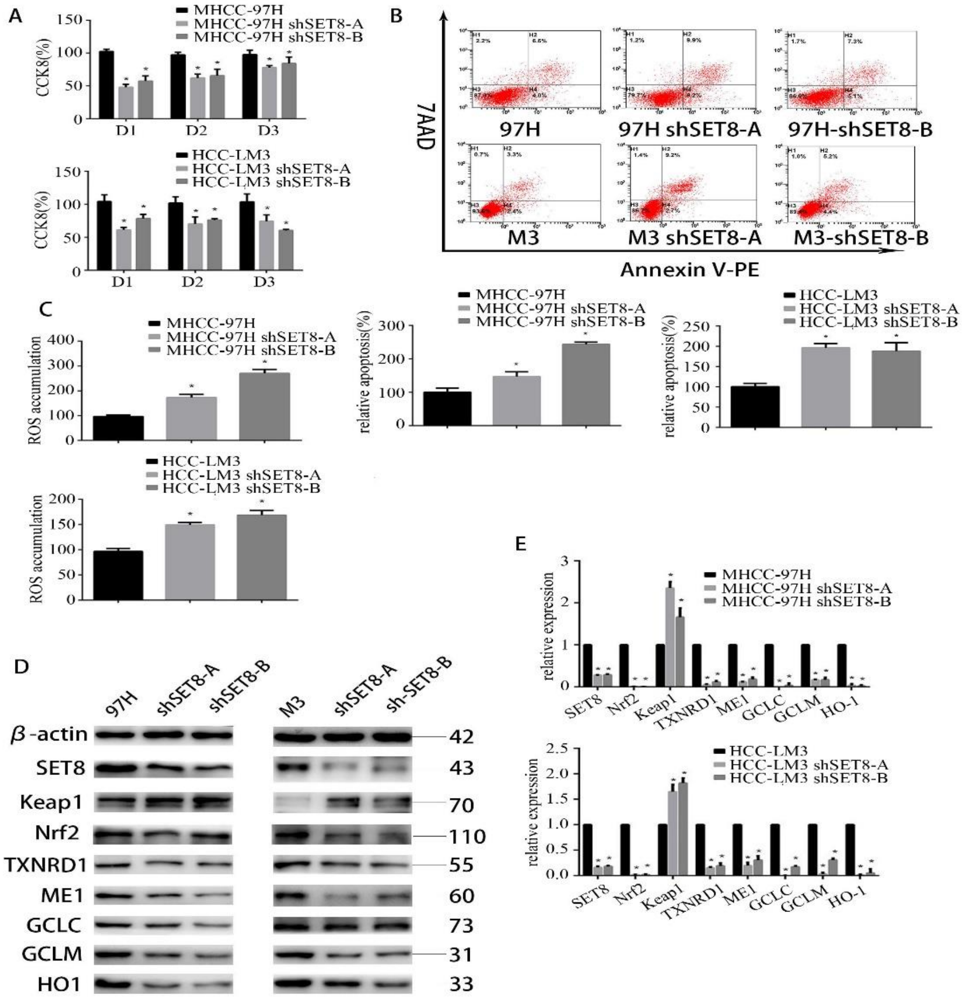

Supplementary Figure S2

(A) Proliferation of MHCC-97H and HCC-LM3 cells following SET8 knockdown. (B) Flow cytometry was used to detect the number of apoptotic MHCC-97H and HCC-LM3 cells following SET8 knockdown. (C) The level of reactive oxygen species in MHCC-97H and HCC-LM3 cells following SET8 knockdown. (D) Western blot analysis of SET8 and Keap1/Nrf2/ARE signalling pathway components in MHCC-97H and HCC-LM3 cells following SET8 knockdown. (E) qPCR analysis of SET8 and Keap1/Nrf2/ARE signalling pathway components in MHCC-97H and

HCC-LM3 cells following SET8 knockdown. Data are shown as the mean ± SD of five independent experiments. *$P < 0.05$ vs the control group.

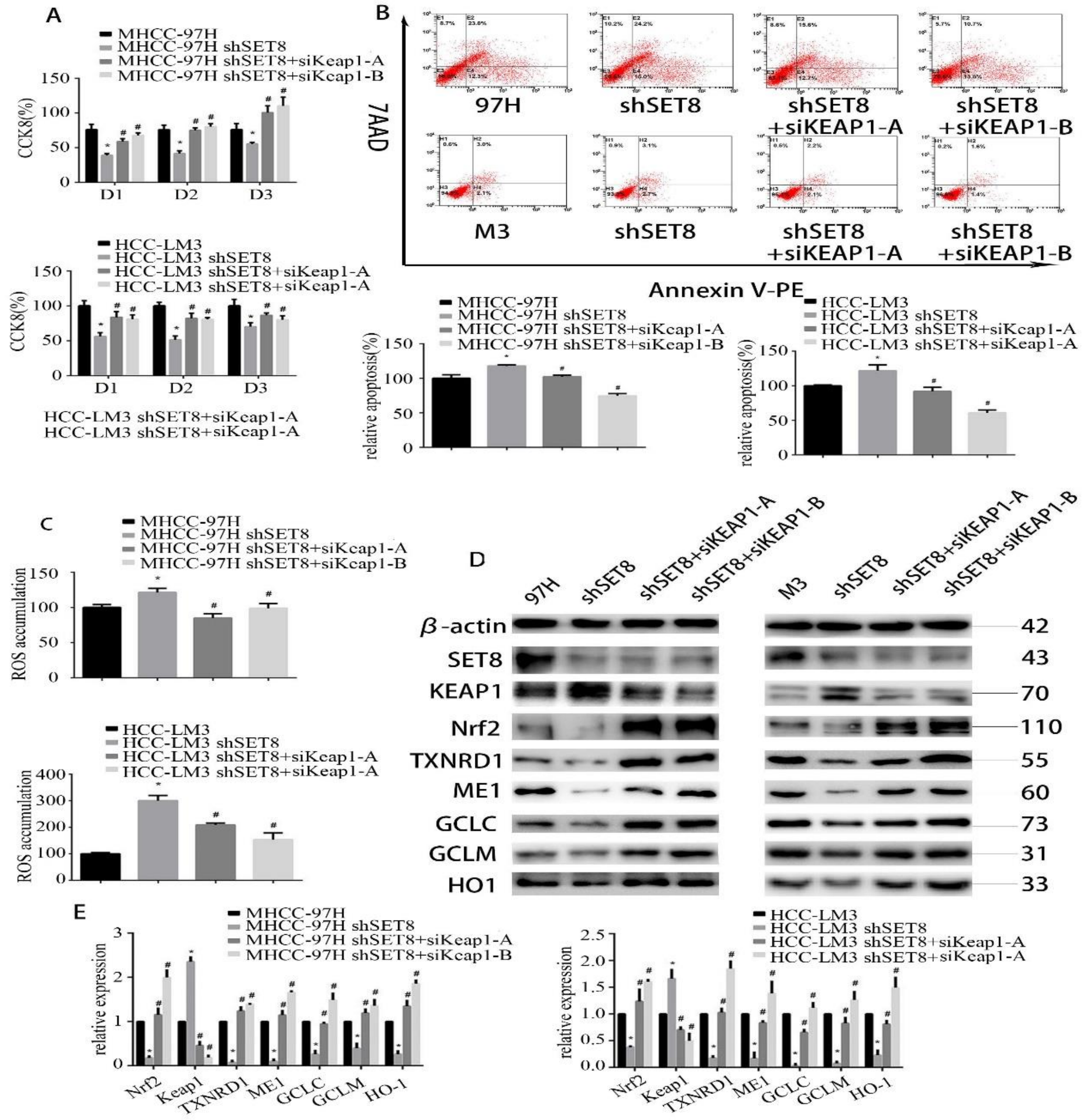

Supplementary Figure S3

(A) Proliferation of MHCC-97H and HCC-LM3 cells following SET8 knockdown alone or in combination with Keap1 knockdown. (B) Flow cytometry was used to detect the number of apoptotic MHCC-97H and HCC-LM3 cells following SET8 knockdown alone or in combination with Keap1 knockdown. (C) The level of reactive oxygen species in MHCC-

97H and HCC-LM3 cells following SET8 knockdown alone or in combination with Keap1 knockdown. (D) Western blot analysis of SET8 and Keap1/Nrf2/ARE signalling pathway components in MHCC-97H and HCC-LM3 cells following SET8 knockdown alone or in combination with Keap1 knockdown. (E) qPCR analysis of SET8 and Keap1/Nrf2/ARE signalling pathway components in MHCC-97H and HCC-LM3 cells following SET8 knockdown alone or in combination with Keap1 knockdown. Data are shown as the mean ± SD of five independent experiments. *P < 0.05 vs the control group. # P<0.05 vs SET8 knockdown group.

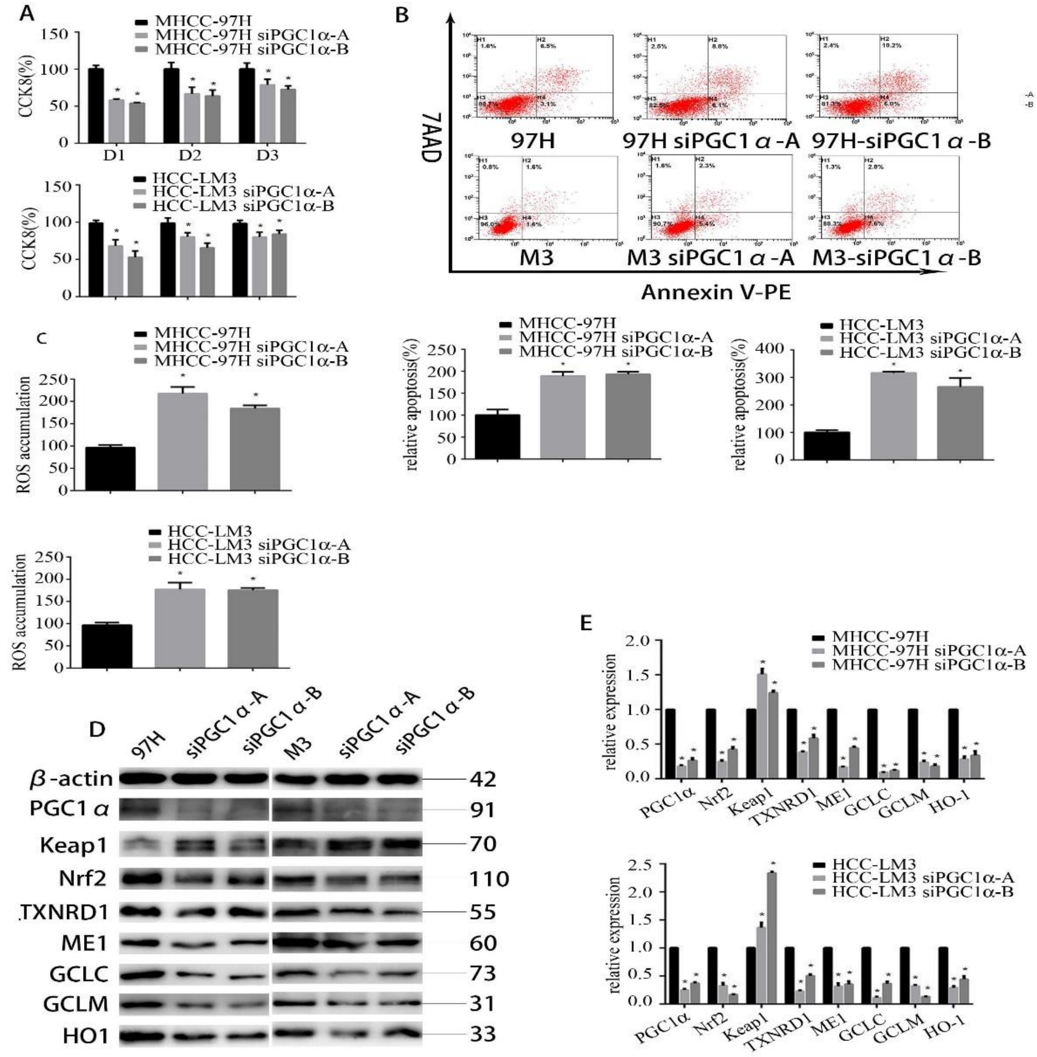

Supplementary Figure S4

(A) Proliferation of MHCC-97H and HCC-LM3 cells following PGC1α knockdown. (B) Flow cytometry was used to detect the number of apoptotic MHCC-97H and HCC-LM3 cells following PGC1α knockdown. (C) The level of reactive oxygen species in MHCC-97H and HCC-LM3 cells following PGC1α knockdown. (D) Western blot analysis of PGC1α and Keap1/Nrf2/ARE signalling pathway components in MHCC-97H and HCC-LM3 cells following PGC1α knockdown. (E) qPCR analysis of PGC1α and Keap1/Nrf2/ARE signalling pathway components in MHCC-

97H and HCC-LM3 cells following PGC1α knockdown. Data are shown as the mean ± SD of three independent experiments. $*P < 0.05$ vs the control group.
